# Motor Cortex Coverage Predicts Signal Strength of a Stentrode Endovascular Brain-Computer Interface

**DOI:** 10.1101/2025.09.19.25335875

**Published:** 2025-09-25

**Authors:** Hunter R. Schone, Peter Yoo, Adam Fry, Nikole Chetty, Abbey Sawyer, Cara Herbers, Fang Liu, Chan Hong Moon, Katya Hill, Shahram Majidi, Noam Y. Harel, Raul G. Nogueira, Elad Levy, David F. Putrino, David Lacomis, Thomas J. Oxley, Douglas J. Weber, Jennifer L. Collinger

**Affiliations:** 1 Rehab Neural Engineering Labs, University of Pittsburgh, Pittsburgh, PA, USA; 2 Department of Physical Medicine and Rehabilitation, University of Pittsburgh, Pittsburgh, PA, USA; 3 Synchron, Inc., New York City, NY, USA; 4 Department of Mechanical Engineering, Carnegie Mellon University, Pittsburgh, PA, USA; 5 Icahn School of Medicine at Mount Sinai Hospital, New York City, NY, USA; 6 Department of Radiology, University of Pittsburgh, Pittsburgh, PA, USA; 7 School of Health and Rehabilitation Sciences, University of Pittsburgh, PA, USA; 8 University of Pittsburgh Medical Center, Pittsburgh, PA, USA; 9 Departments of Neurosurgery and Radiology, University of Buffalo, Buffalo, NY, USA; 10 Departments of Neurology and Pathology, University of Pittsburgh School of Medicine, Pittsburgh, PA, USA; 11 Royal Melbourne Hospital, University of Melbourne, Melbourne, AUS; 12 Department of Bioengineering, University of Pittsburgh, Pittsburgh, PA, USA

## Abstract

Brain-computer interfaces (BCIs) are an emerging assistive technology for individuals with motor impairments, enabling the command of digital devices using neural signals. The Stentrode BCI is an implant, positioned within the brain’s neurovasculature, that can record movement-related electrocortical activity. Over 5 years, 10 participants (8 amyotrophic lateral sclerosis, 1 primary lateral sclerosis, 1 brainstem stroke) have been implanted with a Stentrode BCI and significant inter-participant variability has been observed in the recorded motor signal strength. This variability warrants a critical investigation to characterize potential predictors of signal strength to promote more successful BCI control in future participants. Therefore, we investigated the relationship between Stentrode BCI motor signal strength and a variety of user-specific factors: (1) clinical status, (2) pre-implant functional activity, (3) peri-implant neuroanatomy, (4) peri-implant neurovasculature, and (5) Stentrode device integrity. Data from 10 implanted participants, including clinical demographics, pre- and post-implant neuroimaging and longitudinal Stentrode BCI motor signal assessments were acquired over a year. Across all potential predictors, the strongest predictor of Stentrode motor signal strength was the degree to which the Stentrode BCI’s deployment position overlapped with primary motor cortex (M1). These findings highlight the importance of targeting M1 during device deployment and, more generally, provides a scientific framework for investigating the role of user-specific factors on BCI device outcomes.

## Introduction

**Figure 1.**
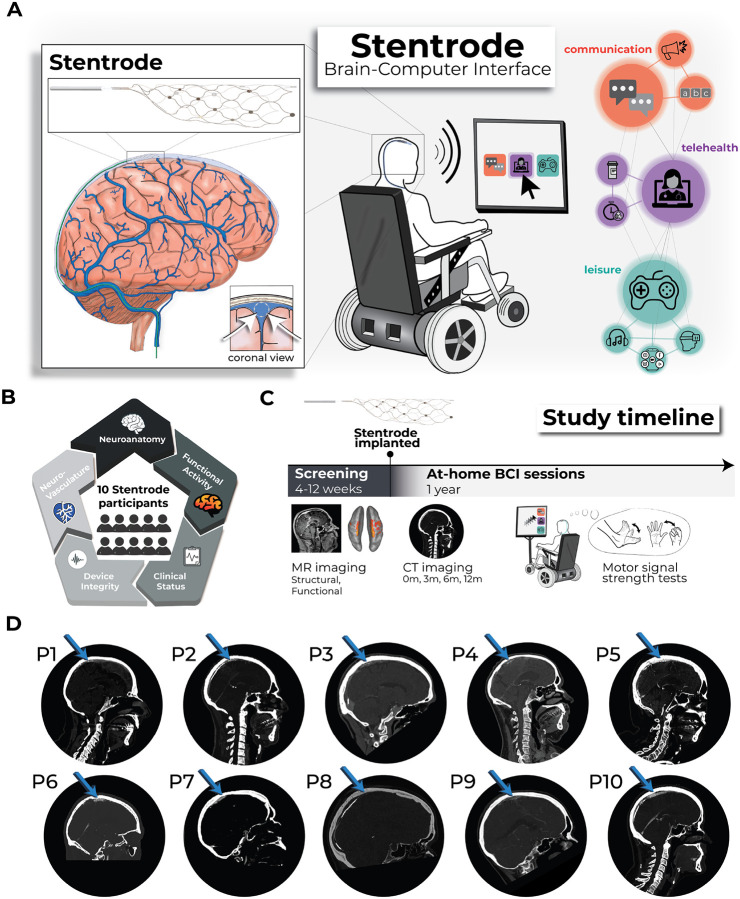
Study overview investigating participants implanted with a Stentrode BCI. **(A) Left –** llustration depicting the Stentrode BCI implanted within the superior saggital sinus. **Middle –** A Stentrode BCI user is depicted generating digital commands with their neural activity. **Right –** Real-world BCI applications include digital communication (orange), telehealth (purple) and leisure (teal). **(B)** Pooling data across 10 participants in the Stentrode BCI clinical trial, we tested which user-specific factors drive Stentrode BCI signal strength, including: neuroanatomy, pre-implant functional MRI activity, clinical condition, device integrity and neurovasculature. **(C)** Clinical trial timeline: pre-implant screening and at-home BCI sessions. **(D)** Post-implant CT scans of each participant at their 3-month timepoint illustrates the relative position of the Stentrode BCI location.

Since the first chronic implant in 1998, brain-computer interfaces (BCI) have steadily emerged as an increasingly feasible class of assistive technology^1^. Typically designed for individuals with motor impairments (e.g., due to spinal cord injury, stroke, or amyotrophic lateral sclerosis), BCIs translate brain signals into digital commands, circumventing impaired peripheral motor pathways to facilitate control of digital and physical end-effectors (e.g., computer cursors, robotic limbs), enable digital communication, and offer social engagement via online platforms^2^. Existing BCI systems can record brain signals at different locations, with systems using electrodes placed on the surface of the scalp or electrodes that interface directly within brain tissue. Each interface site offers distinct trade-offs in signal fidelity, surgical invasiveness, long-term usability and widespread adoption^1^. Scalp electroencephalography BCIs take a noninvasive approach, but suffer from limited signal resolution and prolonged setup times^3^. Subdural electrocorticography (ECoG) makes use of electrodes laid on the surface of the brain and intracortical microelectrode arrays are inserted directly into the cortical tissue. These techniques offer high-resolution neural recordings, but require a craniotomy, i.e., open-brain surgery, thus limiting widespread adoption. Additionally, electrodes implanted directly into brain tissue exhibit a decline in signal quality over time, due to a biological neuroinflammatory response in the brain^4^ and material degradation^5–8^. An emerging alternative interface, the Stentrode BCI (developed by *Synchron, USA*), is an endovascular stent-electrode array that is implanted into the brain’s vasculature using common endovascular procedures. To date, the Stentrode BCI has been placed in the superior sagittal sinus (SSS), a large vein which lies at the midline of the brain, between the hemispheres of the brain. Unlike subdural ECoG and intracortical devices, the deployment approach does not require a craniotomy. Further, by leveraging the location of the neurovasculature relative to the brain, the Stentrode BCI can record distant neural activity without penetrating the cortical surface and is therefore being investigated as an alternative approach to intracortical devices (Figure 1A).

Despite the growing number of BCI technologies, only recently have at-home clinical trials begun to test BCI device usability, marking a critical shift in the focus from proof-of-concept demonstrations in controlled laboratory settings to real-world usability and performance evaluations (for a comprehensive review of all human BCI clinical trials^9^). Industry-led feasibility trials, such as those for the Stentrode BCI [Stentrode clinical trials (Clinicaltrials.gov): AUS (n=4): NCT03834857^10,11^; USA (n=6): NCT05035823], have investigated the safety of the implanted device and provided an opportunity to gather efficacy data, beginning to define for the field what BCI usability looks like in users’ daily lives. However, experience from at-home use of the Stentrode BCI—and similar observations from other BCI technologies—has highlighted a new challenge: BCI signal strength can vary substantially across participants^12–15^. Moving forward, it is necessary to identify which user-specific factors most reliably predict BCI signal strength for each device, whether related to a participant’s clinical status, device design, or where the BCI is implanted. This information is critical for designing pivotal clinical trials that drive the technology’s path to widespread clinical use. Further, considering the clinical risks for any BCI implantation procedure and the costs and resources required to implant a single BCI device, it is necessary to critically evaluate, at this stage, whether any user-specific factors can predict the inter-participant variability in Stentrode BCI motor signal strength. Crucially, this question–what factors best predict successful BCI use–extends beyond endovascular BCIs to the entire field, where predicting and optimizing BCI performance is going to be essential for the widespread clinical deployment of devices and selecting the best BCI technology for each patient’s neuroanatomy, clinical status and functional needs.

In the present work, we aimed to identify factors that may contribute to neural signal strength recorded in individuals who had been implanted with a Stentrode device. We hypothesize that several factors will contribute to the inter-participant variability in BCI motor signal strength, which we define as the amount of modulation in neural recordings during attempted movements^14^. Some of these user-specific factors are unique to endovascular BCIs and others are more general (Figure 1B). These include ***clinical status***, such as time since diagnosis and severity of motor impairment; ***pre-implant cortical function***, reflecting the extent to which users can activate motor cortex during attempted movements as measured by functional neuroimaging; ***peri-implant neuroanatomy***, encompassing the spatial proximity of the implant to cortical tissue and specific brain regions; ***peri-implant neurovasculature***, such as width of the SSS and its relative distance to the cortical surface; and ***device integrity***, specifically the number of active recording electrodes (Figure 1C). To address these open questions, across all participants historically implanted with a Stentrode BCI, we pooled datasets including clinical demographics, functional and structural neuroimaging (pre- and post-implant), and longitudinal Stentrode BCI motor signal strength recordings (n=10; Figure 1D). Combined, our investigation aimed to identify the strongest user-specific predictors of Stentrode BCI motor signal strength. Across all tested predictors, the most significant predictor of Stentrode BCI motor signal strength was how much the Stentrode BCI overlapped with motor cortex, demonstrating that successful endovascular BCI use critically depends on targeting primary motor cortex—highlighting the importance of precise neuroanatomical targeting for future clinical deployment.

## Results

Our pipeline involved first ***extracting the user-specific factors***, then ***quantifying Stentrode BCI motor signal strength*** during attempted movements across multiple sessions, and finally ***building predictive models*** to test which factors are associated with signal strength.

### Clinical status: Heterogeneity in disease state across study participants

Ten study participants with motor impairment were implanted with a Stentrode BCI. Nine participants were diagnosed with adult-onset motor neuron diseases [8 amyotrophic lateral sclerosis (ALS); 1 primary lateral sclerosis (PLS; P4); mean ± STD; 4.5 ± 2.8 years since diagnosis at the time of consent], and 1 participant (P8) was diagnosed with an arterial ischemic stroke in the brainstem (13.5 years since diagnosis at time of consent; see participant demographics in Supp. Table 1). Participants varied in their functional status at the time of implant (for qualitative descriptions of participant’s functional status, see Supp. Table 1). To quantify motor function, participants were graded on their ability to generate muscle contractions of different body-parts: the fingers, wrist, elbow, shoulder, hip, knee, ankle and toes. Participants varied in their manual muscle testing scores (^16,17^). We hypothesized that the progression of a participant’s motor neuron disease pathology or residual motor strength could be potential predictors of Stentrode BCI motor signal strength.

### Pre-implant functional neuroimaging: Preserved ability to activate primary motor cortex

**Figure 2.**
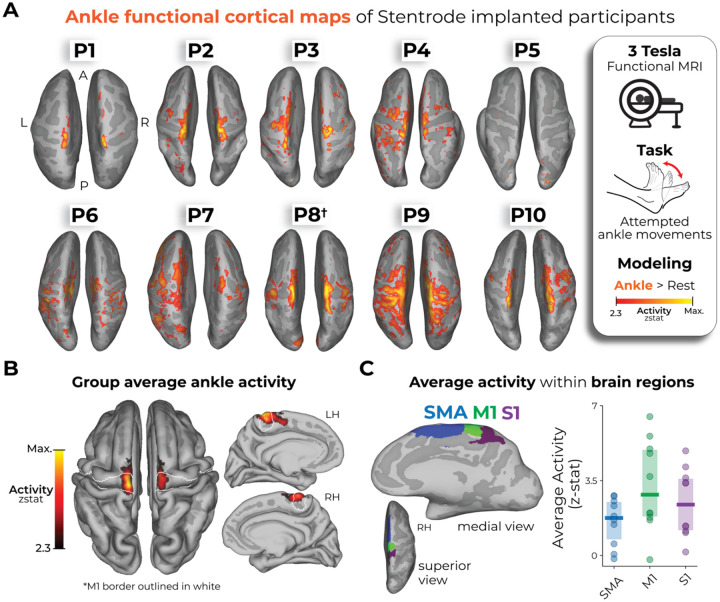
Pre-implant functional neuroimaging of Stentrode-implanted participants. **(A)** Ankle cortical maps of Stentrode-implanted participants. Using a 3 Tesla MRI scanner, participants were cued to perform ankle movements of either a single ankle or both ankles (see Methods). The functional activity for the ankle movement versus rest is projected onto each participant’s inflated cortical surface, depicting just the superior view. The activity is minimally thresholded at a z-statistic of 2.3. All participants were diagnosed with adult motor neuron diseases (ALS or PLS), except for P8 who was diagnosed with a pontine arterial ischemic stroke in the brainstem (depicted with a dagger symbol: †). **(B)** Averaging across participants, the group-level average ankle activation map is projected on a standard pial cortical surface. The primary motor cortex (M1; BA4) boundary is outlined in white. **(C)** Performing an ROI-specific analysis (averaged across both hemispheres), the M1, S1 and SMA ROIs were reduced in size to best capture the relevant ankle activation near the top of the cortical surface, closest to the Stentrode BCI deployment site. **Left** – An example visualization of a participant’s modified ROIs shown on an inflated, right hemisphere (RH) cortical surface. Across participants, M1 exhibited significantly stronger activity than SMA (paired Wilcoxon signed rank test: W=49.0, p_uncorr_=0.02) and S1 (W=47.0, p_uncorr_=0.04). **Right** – The average activity within each ROI for each participant is shown. Blue=SMA; green=M1; purple=S1.

Prior to implantation, all participants underwent functional and structural MRI scans (see Methods). The purpose of the functional MRI was to characterize each participant’s ability to functionally activate sensorimotor cortex during attempted movement. Participants needed to show some significant activation of motor cortex in order to be included in the study. During the functional MRI, participants were cued to perform single- or dual-ankle movements. The ankle was selected because ankle cortical activity is highly medial and superior^18^, making it the closest representation to the planned Stentrode BCI deployment site within the SSS. Projecting the ankle activation maps for each participant onto the cortical surface revealed widespread activation centered around sensorimotor cortices, though varying in breadth and strength across participants (Figure 2A). On average, across participants, the majority of the activity was within the boundaries of M1, somatosensory cortex (S1) and the supplementary motor area (SMA; Figure 2B–C). Further, there were stronger activations in M1 compared to S1 (paired Wilcoxon signed-rank test: W=47.0; *p*=0.04) and SMA (W=49.0; *p*=0.02). However, the spatial spread of significantly activated voxels (Z > 2.3) within each region was largely the same across regions (Supp. Figure 1A; M1 to S1: W=27.0; *p*=1.0; M1 to SMA: W=30; *p*=0.4; S1 to SMA: W=35.0; *p*=0.4).

We note P5 showed minimal functional activation compared to other participants, though there were still significantly activated voxels for ankle movements (see Supp. Figure 1A–B). We suspect that the reduced activation volume can be attributed to either poor task compliance during the scan (e.g., poor adherence to the attempted movement or unintended attempted movement during the rest period) and/or due to the late-stage progression of their ALS pathology (11 years since their diagnosis; see the Discussion section for more information). However, across all participants we saw no significant relationship between a participant’s ability to functionally activate motor cortex (average activation) and years since motor neuron disease diagnosis (*r*_*s*_=−0.50, *p*=0.91). Thus, despite motor neuron disease progression (or P8’s brainstem stroke), all participants were able to functionally activate motor cortex during attempted movement. While there is an obvious potential for selection bias given that the ability to activate motor cortex was part of the inclusion criteria, no participants who were screened were excluded for this reason in either clinical trial. However, some did repeat scans to test for significant activation. Aiming to leverage these preserved cortical motor representations to generate digital commands, all participants were then implanted with a Stentrode BCI (Figure 1D; see Methods).

### Structural neuroimaging: Variability in peri-implant neuroanatomy surrounding the Stentrode BCI

**Figure 3.**
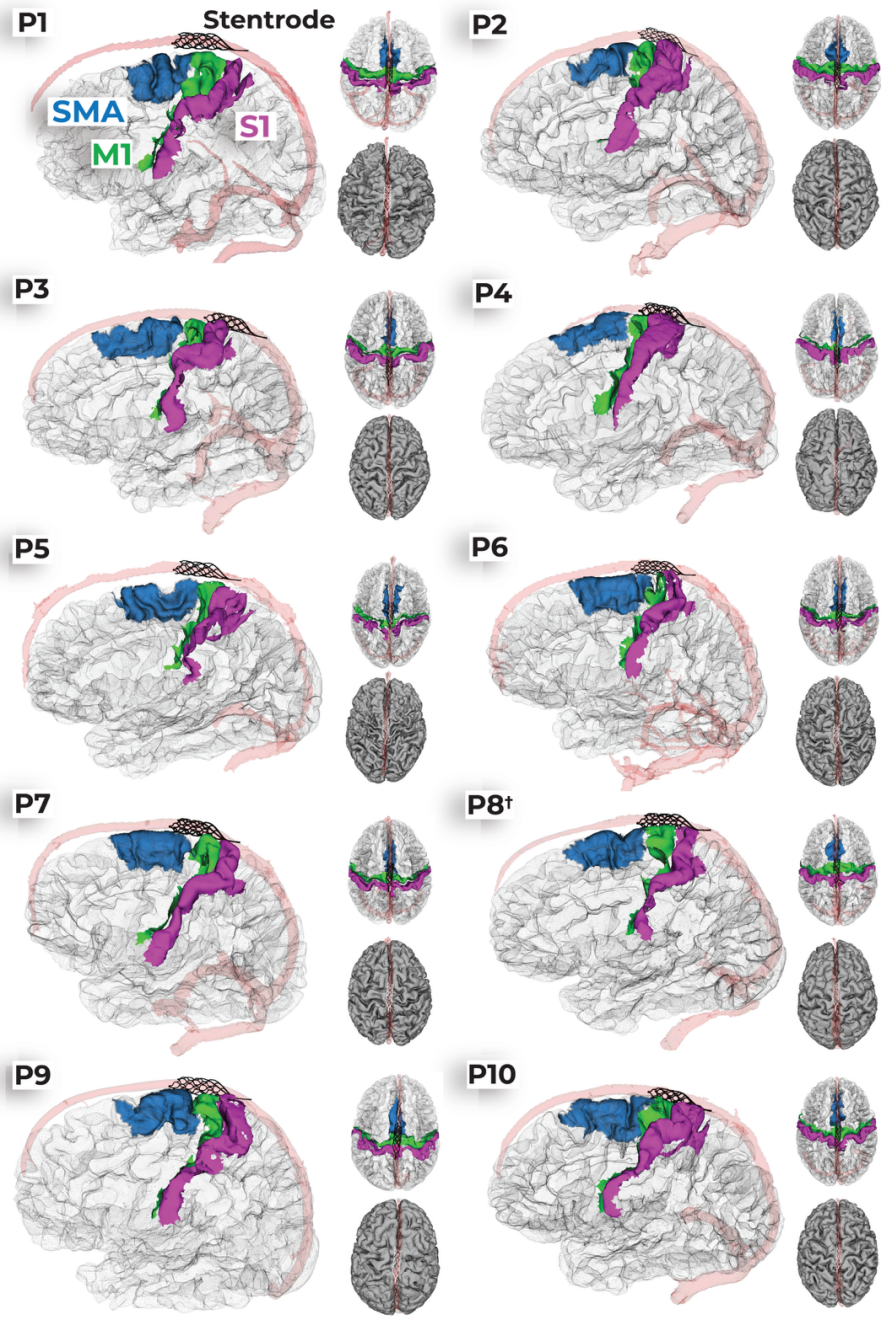
Stentrode BCI position for all participants. For each participant, there are 3 distinct visualizations of the implant position. First, there is a visualization of the sagittal (left) and axial (right top) views of an opaque wire-frame mesh of the cortical surfaces with vertices for specific brain regions colored (supplementary motor area=blue; primary motor cortex=green; somatosensory cortex=pink/purple) and the Stentrode model (black). Second, there is an axial view (right bottom) visualization of each participant’s pial cortical surfaces (grey) depicted with both the superior sagittal sinus segmentation (transparent red) and the Stentrode model (white). P8’s subject label includes a dagger symbol (†) to reflect their brainstem stroke.

Next, we used the pre-implant structural MRI to generate high-resolution images of the cortical anatomy and neurovasculature architecture. After participants were implanted with the Stentrode BCI, CT scans were taken to visualize the location of the Stentrode BCI within the superior sagittal sinus (SSS). Each participant’s 3-month post-implant CT images (Figure 1D) were registered to their pre-implant structural T1w MRI. This allowed for visualization of each participant’s cortical surface reconstruction, neurovasculature and Stentrode BCI segmentations in a common coordinate space (Figure 3; see Supp. Figure 2 for an illustration of the method). Using this aligned data, our goal was to quantify how close the Stentrode BCI was to movement-related neural activity and anatomically-defined cortical regions (i.e., M1, S1, and SMA). We also quantified M1 cortical atrophy for the motor neuron disease participants, since motor neuron disease results in the death of motor neurons as the disease progresses^19,20^.

**Figure 4.**
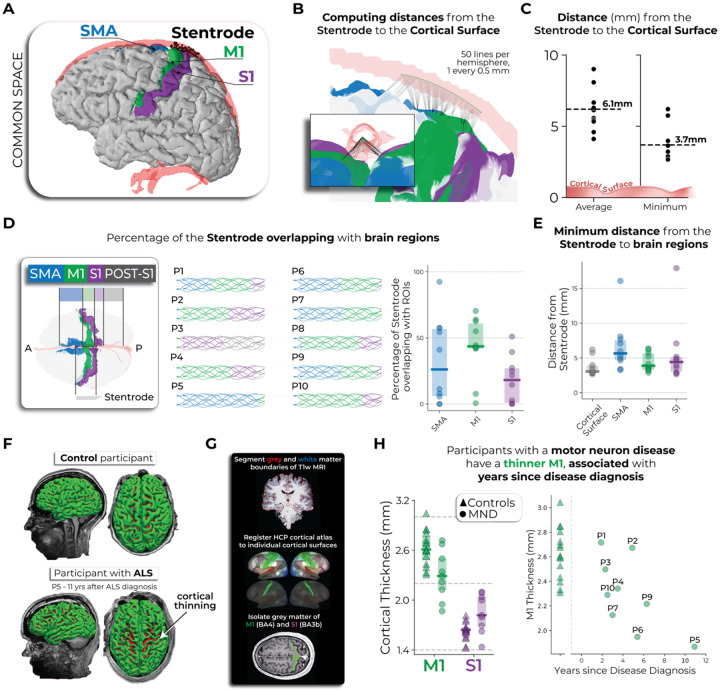
Peri-implant neuroanatomical environment of Stentrode-implanted participants. **(A)** An example participant’s pial cortical surface visualized with their Stentrode segmentation, SSS segmentation, and relevant cortical regions (SMA, M1, S1) registered to the cortical surface. Blue=SMA; green=M1; purple=S1. **(B)** Approximation of the Stentrode implant position in the SSS and its distance to the cortical surface. Black lines indicate shortest distances to cortex. **(C)** The average (data on left) and minimum (data on right) distance from the Stentrode line to the cortical surface is displayed. **(D-E)** Spatial overlap and proximity of each participant’s Stentrode with SMA, M1 and S1. **(F)** Grey matter of a non-ALS control participant (top) and Stentrode-implanted participant with ALS (bottom; 11 years after ALS diagnosis). **(G)** Method for quantifying cortical thickness. **(H) Left** – Stentrode-implanted participants with motor neuron diseases (MND; shown as circles) exhibited a thinner M1, not S1, relative to a group of control participants (shown as triangles). **Right** – M1 cortical thickness was associated with the number of years since each participant’s disease diagnosis, i.e., M1 thins with motor neuron disease progression.

First, we measured the distance between the Stentrode BCI and the cortical surface. Since individual electrodes are embedded at different points around the circumference of the stent scaffold—some closer to the cortex and others within the opposite wall of the SSS—we created a simplified approximation of its location. Specifically, we generated a 25mm-long line from the most rostral point of the Stentrode CT segmentation, extending caudally, centered within the SSS, and shaped to match its inferior curvature (Figure 4A–B). This approximation was used for all subsequent analyses. To account for cortical folding beneath the vasculature, we calculated (1) the average Stentrode-to-cortex distance across hemispheres and (2) the absolute minimum distance. Across participants, the Stentrode was, on average, 6.1 ± 1.5mm from the cortical surface, with the minimum distance of 3.7mm ± 1.2 (averaged across participants; Figure 4C). Though, because the array was modelled in the middle of the SSS, the reported values describe the mean electrode-to-cortex separation across the entire Stentrode, rather than the smallest possible clearance of its nearest contact. In addition to measuring distances to cortex, we quantified the width of the SSS vasculature segmentation around the Stentrode implant site, which averaged 7.1 ± 0.9 mm across participants (Supp. Figure 3).

We next examined the extent to which the Stentrode overlapped with specific brain regions: the SMA (blue), M1 (green), S1 (purple), and any territory caudal to S1 (grey; Figure 4D). While each participant had a unique distribution of overlap across these regions, M1 was the predominant area of coverage for most participants, except for P3 and P5 (for a visualization of all participants cortical surfaces and Stentrode implant position see Figure 3). We repeated the distance calculations shown in Figure 4C for each ROI and found that across participants, the Stentrode was closest to M1 (Figure 4E). Considering this proximity to M1, one important consideration is each participant’s disease progression, since motor neuron disease pathology triggers motor neuron death and M1 cortical atrophy^21–27^. M1 thinning might increase the distance between the Stentrode and desired cortical motor signals, potentially weakening signal strength. For example, see the cortical anatomy of the most progressed participant with ALS in our cohort (P5: 11 years after diagnosis), relative to a control participant without ALS (Figure 4F). Data from 16 control participants of a similar age were pooled from a pre-existing dataset^28^ (age of participants with motor neuron diseases vs. control participants: Mann-Whitney independent samples test: W=109.0, *p*=0.13). To assess whether cortical thinning was observed among Stentrode-implanted participants with motor neuron diseases, we segmented the grey matter of M1 and S1 for each participant and computed the average thickness of each (Figure 4G). We observed that participants with motor neuron diseases (P8 excluded) exhibited a thinner M1, but not S1, relative to control participants [n=16; Figure 4H; rmANOVA: ROI*group interaction: F_(1,21)=_19.6,*p*<0.001; Mann Whitney U tests: M1: W=101, *p*_*corr*_=0.03; S1: W=40, *p*_*corr*_=0.318]. Further, M1 thickness was significantly associated with the number of years since their motor neuron disease diagnosis, i.e., participants that were more progressed exhibited a thinner M1 (Spearman correlation: *r*_*s*_=−0.70, *p*=0.036). Further, muscle strength scores were also significantly associated with the cortical thickness of M1 (*r*_*s*_=0.76, *p*=0.02), and not S1 (*r*_*s*_=0.13, *p*=0.74), such that participants with less residual muscle strength also exhibited a thinner M1. Combined, the analyses highlight the inter-participant variability in the peri-implant neuroanatomical environment that could potentially contribute to Stentrode signal strength.

### Device integrity: Variability in the number of active recording channels across participants

The Stentrode BCI has 16 recording channels that are connected to a intravascular lead that runs through the vasculature, from the SSS to the internal jugular vein. Following Stentrode deployment, the lead was inserted into an inductively powered internal telemetry unit (ITU*; Synchron, USA*) positioned in the chest. Due to the difficulty of ensuring a perfect fit, this procedure can lead to some channels having an inactive connection. As such, across participants, the number of active recording channels varies (9 to 16), which could potentially impact Stentrode motor signal strength, particularly if the inactive electrodes were those that would have recorded the most selective movement-related information.

Due to a technical challenge—and because the participant was the cohort’s only stroke participant, whereas all other participants had ALS or PLS—we excluded P8 from analyses comparing the user-specific factors to Stentrode motor signal strength.

### Stentrode motor signal strength: Detecting attempted movement using the Stentrode BCI

**Figure 5.**
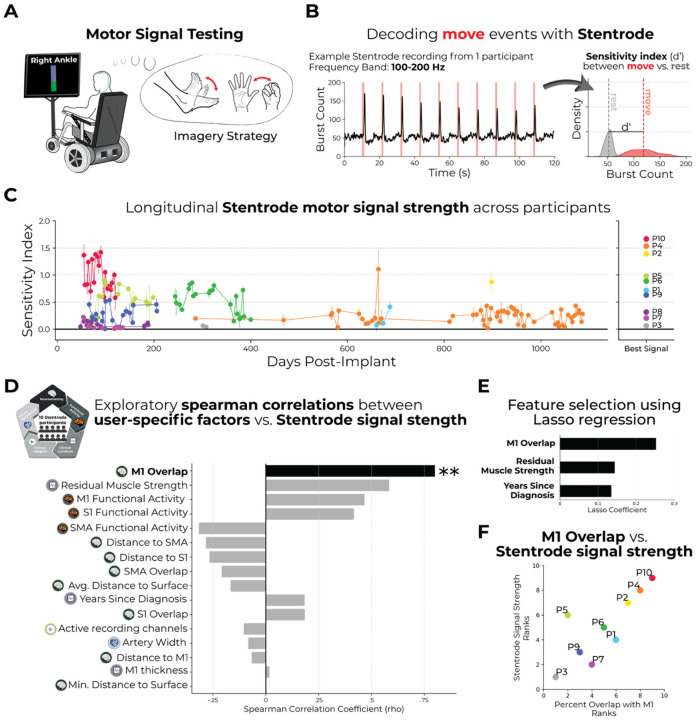
M1 overlap associated with Stentrode BCI motor signal strength. **(A)** To quantify Stentrode BCI signal strength for each participant, participants were visually cued to perform 2 different motor imagery strategies: either an ankle flexion/extension or hand close/open movement (unilateral or bilateral). **(B) Left** – Example Stentrode BCI recording (100–200 Hz frequency band) for 1 participant for a single task session. The red line denotes the on/off period of the movement cue. The black line depicts the Stentrode BCI burst count during the task. **Right** – A signal sensitivity index (d-prime) was computed by comparing burst count densities of ‘rest’ epoched data (shown in grey) versus ‘move’ epoched data (shown in red). **(C) Left** – Longitudinal Stentrode BCI signal strength (i.e., the sensitivity index) values are shown for all participants for all testing sessions. The datasets reflect only the blocks where participants used their preferred imagery strategy. The error bars reflect instances where the task was performed multiple times in a single session. **Right** – The highest Stentrode BCI signal strength value for a single session of each participant are plotted. Participants with the highest session signal strength values are coloured in warm colors and lower in cool colors. **(D)** When correlating Stentrode BCI signal strength to each of the user-specific factors, one significant predictor emerged: percentage of the Stentrode BCI overlapping with M1. **(E)** Similarly, a Lasso regression analysis revealed 3 factors with non-zero lasso coefficients: M1 overlap, residual muscle strength and years since diagnosis. **(F)** Visualization of the correlation between the percentage of the Stentrode BCI overlapping with M1 and motor signal strength (r_s_=0.80, p_uncorr_=0.01; ranked values shown in correlation).

With multiple user-specific factors computed, we next aimed to compute a single measure of Stentrode motor signal strength for each participant. Crucially, we opted to compute a signal strength measure, as opposed to a BCI performance measure due to changes in decoders and electrode referencing across participants and clinical trials. To compute this measure, participants routinely performed motor signal testing (see ref^14^), where participants were visually-cued to attempt either hand or foot movements to generate a BCI click within 10 seconds, followed by resting (no movement) for 10 seconds (10 repetitions per task run; Figure 5A). Considering this test was performed within the context of the larger clinical trials and procedures steadily evolved over time, it is worth noting the Motor Signal Test was not administered to all participants in the same manner. These inconsistencies introduce variability in the data collected, including differences in the number of blocks performed per session, total sessions per participant, and the type of imagery strategy attempted (hand or ankle; unilateral or bilateral). Though, to be consistent across participants, we focused on datasets when participant’s used their preferred imagery strategy (n=3 hand-based; n=7 ankle-based; Supp. Table 1; for data of all imagery strategies see Supp. Figure 4). For each test block, we isolated the high-gamma frequency band (100 – 200 Hz) across all Stentrode channel recordings and computed a sensitivity index between the density of the burst count during the move epochs versus the rest epochs (see Methods; Figure 5B). Hereafter, we refer to this sensitivity index measure as Stentrode motor signal strength^14^.

For each participant across testing sessions, Stentrode motor signal strength values were significantly greater than 0 (one Wilcoxon signed-rank test per participant; 0.0001 < *p* < 0.01; P3 was not included in the group analysis due to only having 2 sessions of this task, though both were greater than 0), where a value of 0 reflects no difference in Stentrode recordings during move events and rest events. Across participants and sessions, we observe variability in the magnitude of Stentrode motor signal strength (Figure 5C). To generate a single value for each participant’s Stentrode motor signal strength to compare to the user-specific factors, we opted to select each participant’s best signal strength value captured in any given test block. The rationale for this decision was to mitigate the inconsistencies in how the task was administered between clinical trials and the progressive nature of motor neuron disease. We note that P4’s best session appears to be an outlier relative to the other sessions (Figure 5C), however P4 had similarly high values in other sessions when using their non-preferred imagery strategy (Supp. Figure 4).

### Statistical comparisons: M1 overlap best predicts Stentrode motor signal strength

Using this measure of Stentrode motor signal strength, we next performed exploratory Spearman correlations to the multiple user-specific factors extracted (16 tests total). Across all tests, we identified only one significant predictor: the percentage of the Stentrode overlapping with M1 (*r*_*s*_=0.80, *p*_*uncorr*_=0.01), such that greater M1 overlap reflects increased Stentrode motor signal strength (Figure 5D; for a visualization of all ranked correlations see Supp. Figure 5). To explore the relationships between Stentrode signal strength and the user-specific factors more rigorously, we also performed a feature selection using Lasso regression. This analysis allowed us to identify which factors were most predictive of Stentrode signal strength while penalizing irrelevant or redundant predictors. Among all factors considered, three factors survived, with greater than zero Lasso coefficients: M1 overlap (the highest), followed by residual muscle strength, and years since diagnosis (Figure 5E). Collectively, these tests show the amount of the Stentrode overlapping with motor cortex is the strongest predictor of Stentrode motor signal strength with clinical measures such as residual muscle strength and disease duration contributing additional, complementary explanatory power when considered in combination. (Figure 5F).

### Considerations for future Stentrode BCI deployment

**Figure 6.**
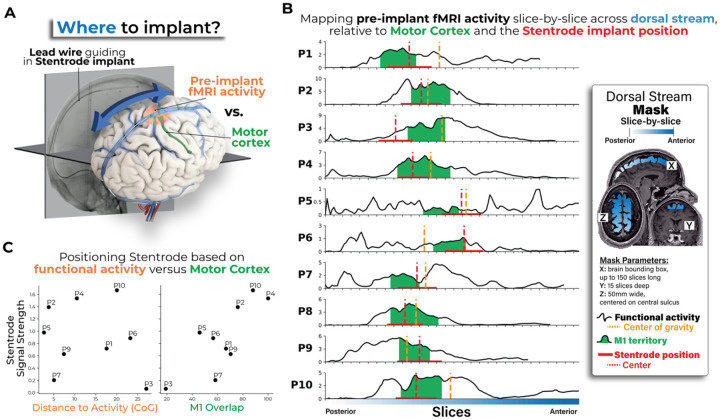
Motor cortex coverage, over functional activity, is strongly associated with Stentrode BCI signal strength. **(A)** An important consideration when deploying the Stentrode BCI via a lead wire is deciding where to position the device within the superior sagittal sinus. There are two factors to base this decision on: (1) pre-implant fMRI activity, relying on the location of the largest cluster of activity or (2) neuroanatomical landmarks, i.e., motor cortex on the rostral wall of the central sulcus. **(B)** To test which factor impacted signal strength in the participants, we performed a volume-based analysis. For each participant (each row), we projected the average pre-implant fMRI motor activity (black line) for each slice across the dorsal stream (white-to-blue colored mask). We then computed the center of gravity of this projection across the dorsal stream, depicted as a yellow dashed vertical line. This would reflect the best implant site if based solely on the fMRI activity. We next defined the slices that overlap with the M1 cortical region (colored in green) and the slices that overlap with the Stentrode BCI (red horizontal line; the center point depicted as a red dashed vertical line). **(C)** Finally, for each participant, we then computed (1) the distance between the fMRI center of gravity slice to the Stentrode BCI center slice and (2) the percentage of Stentrode slices overlapping with M1 slices, i.e., M1 coverage. We observed that M1 coverage showed a significant association with Stentrode signal strength (Spearman correlation r_s_=0.70, p=0.043). Alternatively, there was no meaningful relationship with the distance to the ideal fMRI slice and Stentrode BCI signal strength (r_s_=−0.18, p=0.64).

The previous analyses (Figure 5F) show that M1 overlap is associated with Stentrode BCI signal strength. For future Stentrode implant targeting, this result suggests that deployment should specifically target primary motor cortex, defined by its neuroanatomical landmarks, e.g., the rostral wall of the central sulcus, to maximize Stentrode BCI signal outcomes. However, it is still unclear whether pre-implant fMRI activity can be used to further optimize positioning (Figure 6A). To test this, we aimed to directly compare volume-based fMRI activity versus M1 overlap. We performed a volume-based fMRI activity analysis, where we projected the average pre-implant fMRI motor activity for every slice across the dorsal stream, i.e., the cortical surface directly beneath the SSS vasculature. To select the ideal implant location based on the fMRI activity, we computed the center of gravity of this projection. We next identified, for each participant, the slices that overlap with their Stentrode BCI and the M1 brain region. Finally, we computed the distance between the ideal fMRI slice (i.e., center of gravity of movement-related activity) and the slice at the midpoint of where the Stentrode BCI was implanted (Figure 6B). Often the centroid of activity was rostral to the midpoint of the Stentrode. For five out of ten participants, the centroid of activity was within M1; for four participants the centroid was rostral to M1 and for one participant it was caudal to M1. We also computed the percentage of slices that encompassed the Stentrode BCI that overlapped with M1 (Figure 6B). Similar to the surface-based analysis, we observed that M1 overlap, defined on the volume vs. on the surface, also showed a significant association with Stentrode BCI signal strength (Spearman correlation: *r*_*s*_=0.70, *p*=0.043). Alternatively, there was no meaningful relationship between Stentrode signal strength and how far away the Stentrode BCI was from the location of peak fMRI activity (*r*_*s*_=−0.18, *p*=0.64; Figure 6C). Combined, with the previous analyses, this confirms future Stentrode BCI deployment should prioritize maximizing motor cortex coverage and the present functional MR imaging protocol may be insufficient for improving targeting.

## Discussion

Across 10 participants implanted with a Stentrode BCI, we explored the relationship between a variety of user-specific factors and Stentrode BCI motor signal strength. Regardless of impairment level or diagnosis (ALS, PLS or brainstem stroke), participants were able to functionally activate cortical motor networks during attempted movement prior to implantation, confirming that the ability to voluntarily produce cortical motor signals were preserved in the cohort, at least to some degree. Structurally, we observed that the study participants with motor neuron diseases exhibited motor cortical atrophy relative to controls, with cortical thickness correlating with both participants’ residual muscle strength and time since disease diagnosis. Additionally, we quantified the inter-participant variability of the peri-implant neural environment across all participants implanted with a Stentrode BCI, relative to specific cortical regions and the neurovasculature. Most importantly, across all tested user-specific factors, we identified that the strongest predictor of BCI motor signal strength was the degree to which the Stentrode implant overlapped with primary motor cortex (M1), emphasizing the importance of precisely targeting M1 during device deployment. Our findings have three main implications for clinical neuroscience research and the development of BCI technologies. Specifically, it (1) highlights the unique neural and clinical considerations for BCI signal strength for individuals living with adult-onset motor neuron diseases, (2) informs the future targeting strategy for Stentrode BCI deployment (and other future BCIs that may use an endovascular electrode deployment approach) and, (3) offers a general investigational framework for testing the impact of user-specific factors on signal strength of BCI technologies.

First, our results offer a high-level characterization of the neural and clinical factors that need to be considered for successful BCI usability in individuals living with adult-onset motor neuron diseases, a primary target clinical population for BCI technologies. Most common in our cohort, ALS is a heterogenous neurodegenerative disease, marked by progressive motor decline and generally poor prognosis; population studies report a median survival of ~3 years from symptom onset in unventilated cohorts^29^, but survival can extend well beyond this in individuals who opt for long-term mechanical ventilation or present with slower-progressing variants, such as the flail-arm phenotype that characterized many participants in the present cohort. For people with ALS to successfully use a Stentrode BCI—or any BCI—it is essential they have some intact structure and function of cortical motor networks, with M1 being the dominant region of interest. However, decades of research has investigated how ALS pathology uniquely degrades M1 micro- and macro-structure and functioning. ALS pathology most commonly first targets lower motor neurons, followed by pathological changes throughout cortex, particularly a degradation of the upper motor neurons in M1^19,20,30^. Though, the symptomatic presentation of ALS is highly heterogeneous. Indeed, M1 is the key hub for voluntary motor command output, and multiple studies have demonstrated its vulnerability in later stages of ALS. For example, post-mortem and high-field neuroimaging studies have highlighted that individual’s with late-stage ALS exhibit multiple pathologies to M1 micro- cortical structure, including the degeneration of Betz pyramidal cells in layer V^30^, the accumulation of iron in deeper cortical layers^19,23^, increased intracellular calcium^31^, and progressive, widespread demyelination, particularly at the boundaries between somatotopic regions^19^. In terms of M1 macrostructure, similar to our findings, several cross-sectional studies have demonstrated that individuals with ALS exhibit dramatic cortical atrophy of the precentral gyrus (i.e., pre- and primary-motor cortex), compared to individuals without ALS^21–27^. For example, in a recent report of a long-term ECoG BCI case study, longitudinal CT imaging data from 1 participant revealed significant fronto-temporal cortical tissue atrophy over an 8-year period^32^. Notably, for our investigation, neither M1 cortical atrophy nor the minimum distance between the Stentrode and motor cortex were significant predictors of Stentrode BCI motor signal strength (Figure 5D). Instead, the significant association we observed was with the percentage of the Stentrode BCI overlapping with M1–a distance measure solely on the coronal plane–suggesting that larger Stentrode BCI motor signal strength may be attributed to the maximum number of Stentrode electrodes being closest to motor cortex versus other cortical regions. Indeed, even though the motor cortex progressively atrophies with disease progression, any space between motor cortex and the vasculature is filled with an expanding gap of cerebrospinal fluid (CSF; Supp. Figure 3). Our results suggest that so long as the overlap with M1 is sufficient, the high electrical signal conductivity of cerebrospinal fluid^33^ may still effectively be delivering a selective motor signal to the Stentrode BCI electrodes. This would suggest that cortical atrophy alone should not be an exclusion criteria for Stentrode BCI usability, especially given prior BCI studies demonstrating successful motor decoding in individuals with ALS^14,32,34–37^.

Next, looking to M1 cortical function, neuroimaging studies have reported mixed findings on whether individuals with ALS—particularly those who are locked-in—can volitionally activate M1 during attempted movements similar to control participants (see ref.^38^ for a comprehensive review of task-based neuroimaging studies of ALS). While some studies, including a recent high-field neuroimaging case report, observed preserved M1 activity during motor tasks^39^, others have shown highly variable or diminished responses, likely reflecting the extreme heterogeneity of ALS^38^. This preservation in cortical motor activity, at least to some degree, could be explained by evidence that people with late-stage ALS show a considerable amount of preservation of the upper motor neurons of M1 output layer V^40–42^, at least sufficient for generating a motor output signal. However, it remains an open question whether the presence or strength of M1 activity pre-implant can be used as a reliable predictor of BCI performance. The intricate relationship between M1 structure and function and clinical status—including disease duration and residual muscle strength—likely contributes to BCI signal strength. While no single clinical measure was individually significant in explaining variability, both residual muscle strength and years since diagnosis were retained as predictors in the Lasso regression model. This suggests that, although their individual effects may be subtle, these clinical factors may contribute meaningful, non-redundant information when considered alongside the implant’s proximity to M1. These findings underscore that successful BCI use in people with ALS is not determined by any single clinical or neural factor, but instead reflects a complex interplay between disease progression, preserved cortical function, and precise anatomical targeting—highlighting the need for multimodal, individualized assessment in guiding future BCI deployment.

The most practical implication of our results is they provide immediate guidance on future endovascular BCI deployment strategies, specifically to what degree functional neuroimaging data should be relied on as a targeting guide versus neuroanatomical landmarks (e.g., M1 located on the rostral wall of the central sulcus). For both surface- and volume-based analyses, Stentrode BCI overlap with M1 emerged as a predictor of Stentrode BCI motor signal strength. Further, across participants, the spatial landscape of pre-implant functional activity during attempted movement often lacked a clear unimodal structure along the coronal plane, with the largest peak appearing in different regions (i.e., S1, M1 or SMA) at the individual participant level (Figure 6). In order for personalized functional neuroimaging to be a useful targeting guide, spatially selective functional activations are essential to identify an optimal deployment site. However, in order to visualize more spatially selective activations, it requires changing the MR imaging protocol to incorporate (1) more functional data (e.g., more than the 1–2 runs per participant collected in the present study), and (2) more movement conditions to control for regions responsive to general motor responses, the presence of visual/auditory stimuli during the active epochs, and attentional/motivational drivers of activity. While there is a simple solution, increasing scan times for people with motor neuron diseases, particularly those in advanced stages, has considerable tradeoffs. For example, in the present cohort, 3 participants were mechanically ventilated via a tracheostomy and had minimal to no residual volitional movement of a single body-part (see Supp. Table 1 for descriptions of each participants’ clinical state). Scanning these participants required participants being moved off their personal ventilators, manually ventilated by a respiratory therapist and then placed on an MR-compatible ventilator for the duration of the scanning, regularly monitored by a respiratory therapist. During the scan, the respiratory team had to ensure participants had a clear airway, requiring monitoring of the participant’s residual eye movements and physiological recordings (e.g., pulse oximeter, EKG, and the ventilator, etc). Therefore, due to burden of longer functional scan times in these participants, our recommended guidance for Stentrode deployment is to prioritize maximizing coverage of M1, specifically the rostral wall of the central sulcus. To the extent that the Stentrode BCI is larger than M1, it may be beneficial to bias the positioning more rostral so as to be able to record activity from SMA as well, given its putative involvement in higher order motor and cognitive processes that may be beneficial for complex BCI control^43,44^. However, while present device deployment may not warrant necessitating functional neuroimaging for targeting, next-generation devices could be engineered to target smaller cortical vessels, where a smaller cortical target is necessary. In these instances, functional neuroimaging protocols will need to be improved to ensure a sufficient amount of functional data is acquired to guide targeting of smaller cortical sites. More generally, for BCI devices with broad cortical coverage (i.e., the Stentrode BCI, larger ECoG arrays), we recommend prioritizing anatomical targeting—specifically M1—over individualized functional imaging, that is unless neuroimaging protocols can be optimized (e.g., increased scan times and conditions) to yield sufficiently selective activations without imposing undue risk or burden on participants with advanced disease.

There are several limitations with our investigation that are worth noting. First, we did not perform any statistical corrections for the multiple comparisons, e.g., the 16 user-specific factors tested (Figure 5D–E). The rationale for this decision was due to the highly exploratory nature of the investigation. Our hope is that this data serves as a proof-of-concept to guide the design of future studies with larger cohorts, as they become available. A second limitation is that our estimation of the Stentrode BCI position was accurate solely along the coronal plane, but not accurate in relation to the unique locations of individual electrodes within the SSS. Due to the methodological difficulties with reliably extracting single electrode positions from the CT images and computing distances for each electrode to various targets, we opted for a simpler estimation that was accurate along the coronal axis and positioned within the center of the SSS segmentation. A third limitation is that we provide a measure of motor BCI signal quality, not BCI performance. Finally, considering the aim of the investigation was to pool data across 2 clinical trials (spanning over 5 years), there are differences in the referencing scheme of the Stentrode BCI and the experimental design of the Motor Signal Test used to estimate Stentrode BCI motor signal strength between the two study cohorts. Despite these differences, we did not observe any differences in Stentrode BCI motor signal strength between participants from the two trials, with a similar percentage of participants from each trial as high-responders (upper-half of the sensitivity index distribution) and low-responders (lower-half of the sensitivity index distribution; independent samples Mann-Whiteny U test; W=13.0; *p*=0.914).

In conclusion, our study provides a comprehensive scientific framework for investigating how a variety of user-specific factors contribute to recorded signal strength of implantable BCI technologies. As more individuals are implanted with BCI devices, identifying key user-specific predictors of BCI success will be essential for the long-term viability of the neurotechnology industry. These predictors will be vital for refining patient selection, optimizing implant targeting strategies and tailoring BCI hardware and software for specific clinical populations. By identifying potential determinants of successful neural interfacing with the Stentrode BCI, we hope this investigation offers some foundational insights into improving the clinical translation of the Stentrode BCI and, more broadly, contribute to the advancement of all implantable BCI technologies.

## Supplementary Material

1

## Figures and Tables

**Table 1.** Participant demographics. Regarding the host country of the clinical trial, the SWITCH trial was conducted in Australia (AUS) and the COMMAND EFS trial was conducted in the USA. Y=yes; N=no; PLS=primary lateral sclerosis; ALS=amyotrophic lateral sclerosis.

*Participant ID*	P1	P2	P3	P4	P5	P6	P7	P8	P9	P10
***Sex***	M	M	M	M	M	M	M	F	F	M
***Disease/injury***	ALS	ALS	ALS	PLS	ALS	ALS	ALS	Pontine arterial ischemic stroke	ALS	Main-in-the-barrel ALS
***Years since diagnosis***, *at time of consent*	1.9y	4.9y	2.3y	3.5y	10.9y	5.4y	3.0y	13.5y	6.3y	2.5y
***Mechanically ventilated***, *during study*	N	N	N	N	Y	Y	Y	N	N	N
***Preferred imagery strategy for Stentrode control***	Left ankle	Both ankles	Right ankle	Both ankles	Both ankles	Right hand	Both ankles	Both ankles	Right hand	Right hand
***Stentrode device version; # of connected (active) channels***	V1;16	V1;15	V1;15	V1; 15	V2;15	V2;9	V2;13	V2;13	V2;12	V2;10

## Data Availability

The data cannot be made publicly available upon publication because they contain commercially sensitive information. The data that supports the primary findings of this study are available upon reasonable request.
