# Supplementary material for: Motor Cortex Coverage Predicts Signal Strength of a Stentrode Endovascular Brain-Computer Interface
Source: medRxiv. 2025 Sep 25:2025.09.19.25335875. Preprint. [Version 1] doi: 10.1101/2025.09.19.25335875 (PMC12486047; doi:10.1101/2025.09.19.25335875)
Supplement: 1 [file NIHPP2025.09.19.25335875V1-supplement-1.pdf]

medRxiv preprint doi: https://doi.org/10.1101/2025.09.19.25335875; this version posted September 25, 2025. The copyright holder for this preprint (which was not certified by peer review) is the author/funder, who has granted medRxiv a license to display the preprint in perpetuity.

It is made available under a CC-BY 4.0 International license .

Supplementary Results

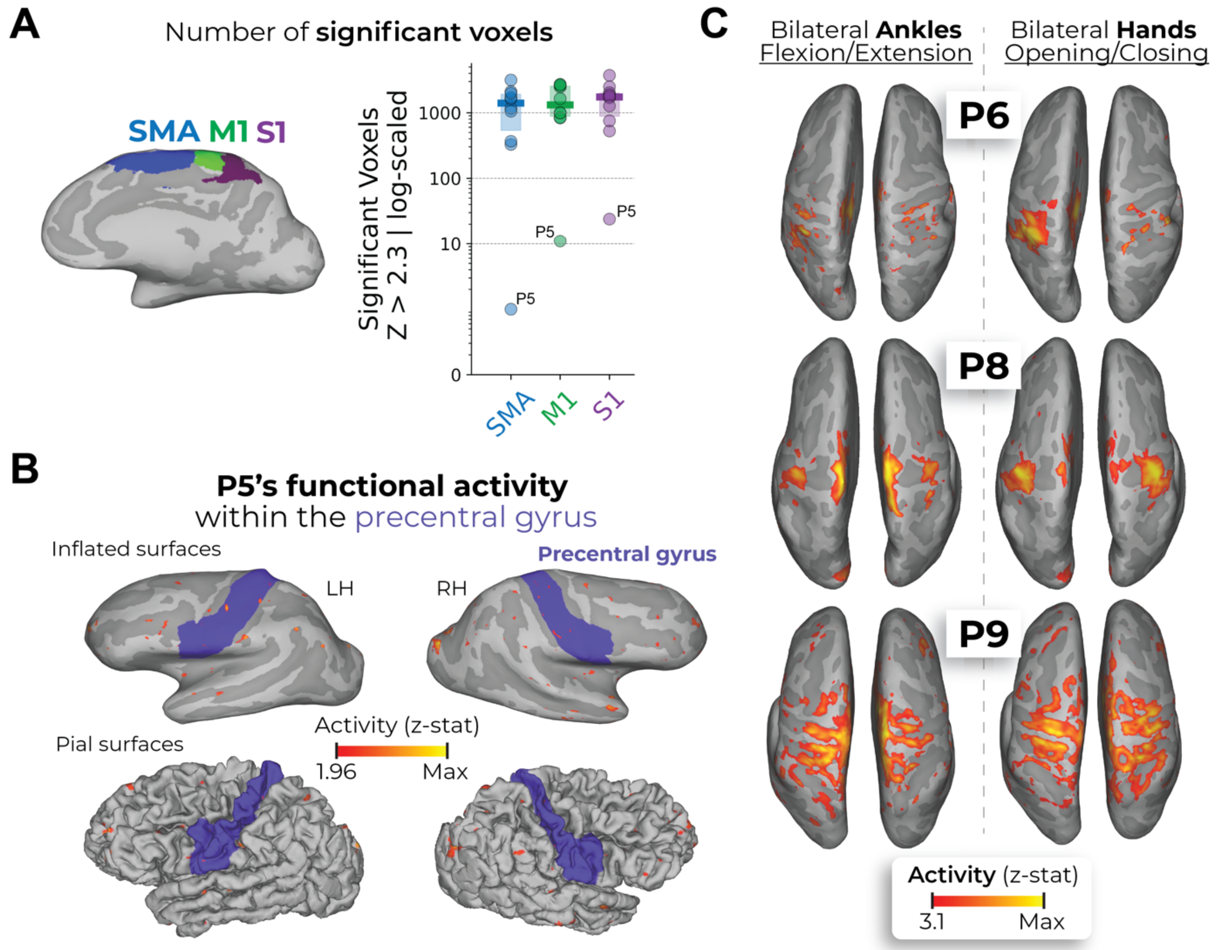

Supp. Figure 1. Supplementary functional neuroimaging analyses. (A) *Number of significant voxels (z-statistic > 2.3; z-threshold used in Figure 2) within the SMA (blue), M1 (green) and S1 (purple) regions. All participants can significantly activate a subset of voxels, within all regions, during attempted movement. The data was visualized on a log-scale to demonstrate that P5 significantly activated a subset of voxels.* ***(B)*** *Visualizing P5's functional data at a lower minimum threshold (z-statistic > 1.96) within the precentral gyrus highlighted (in blue) on both the inflated surfaces (top row) and pial surfaces (bottom row).* ***(C)*** *A subset of participants underwent functional scans where bilateral hand movements were performed. The bilateral ankle and bilateral hand activation maps are displayed. All other annotations are the same as described in Figure 2.*

medRxiv preprint doi: https://doi.org/10.1101/2025.09.19.25335875; this version posted September 25, 2025. The copyright holder for this preprint (which was not certified by peer review) is the author/funder, who has granted medRxiv a license to display the preprint in perpetuity.

It is made available under a CC-BY 4.0 International license .

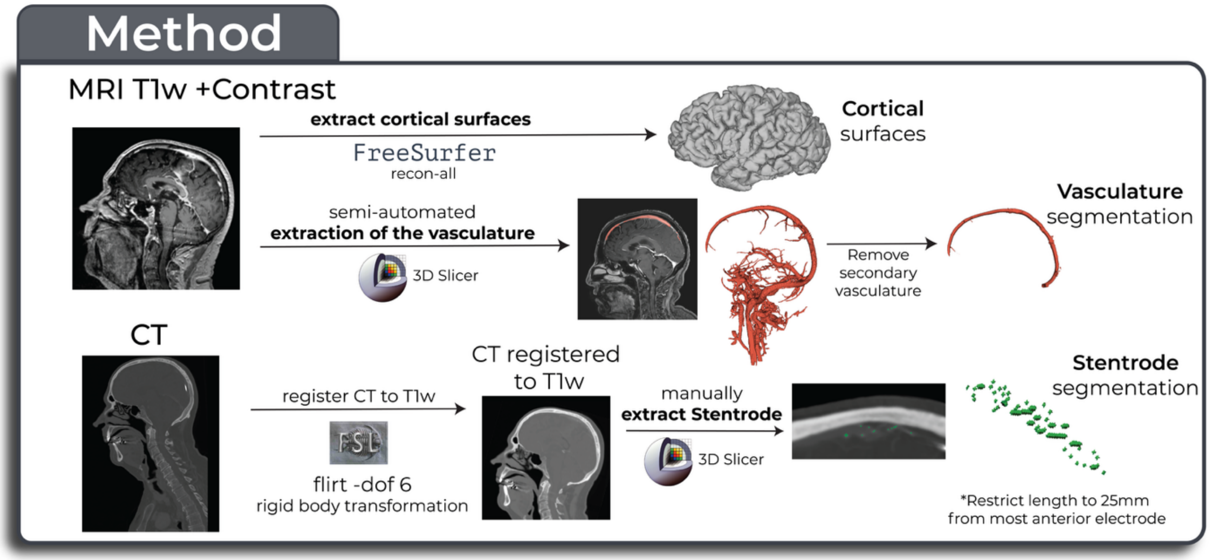

Supp. Figure 2. Method for generating a common coordinate space with the cortical surfaces, vasculature and Stentrode BCI. *See the Methods for a detailed description of the method.*

medRxiv preprint doi: https://doi.org/10.1101/2025.09.19.25335875; this version posted September 25, 2025. The copyright holder for this preprint (which was not certified by peer review) is the author/funder, who has granted medRxiv a license to display the preprint in perpetuity.

It is made available under a CC-BY 4.0 International license .

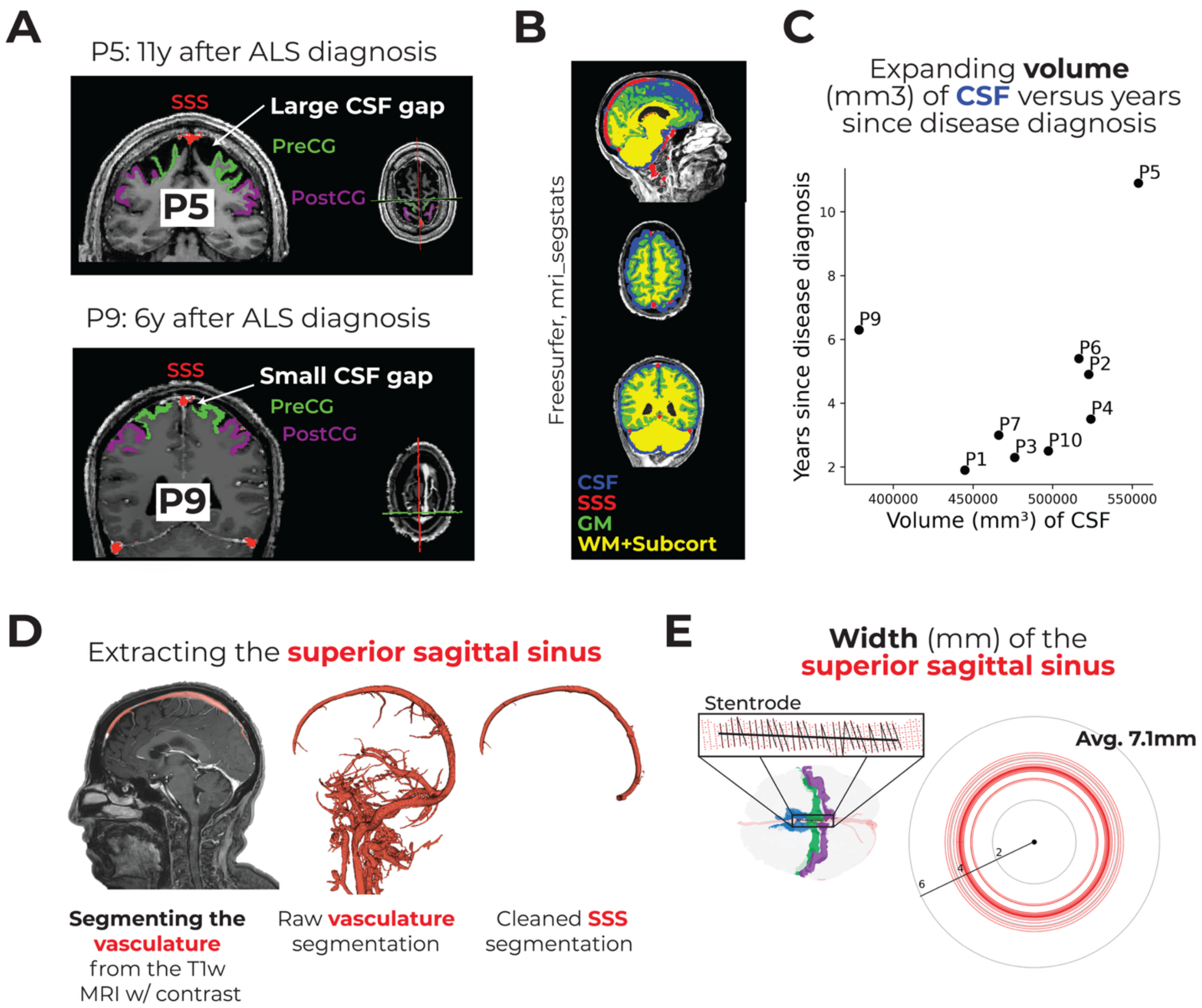

Supp. Figure 3. Segmenting the cerebrospinal fluid (CSF) and superior sagittal sinus (SSS) for Stentrode-implanted participants with adult-onset motor neuron diseases. (A) *Visualizing the SSS, pre- and post-central gyri on the T1w + contrast MRI for the two participants with the most progressed ALS: P5 (top row) and P9 (bottom row). P5 shows an extensive gap, in this extreme slice example, between the SSS and the cortical surface filled with CSF. Alternatively, P9 generally shows a much more minimal gap.* ***(B)*** *To segment the CSF, we used freesurfer's mri_segstats function to generate segmentations of the CSF (blue), grey matter (GM; green), white matter and subcortical structures (both shown in yellow). As the CSF segmentation includes the SSS, we removed any SSS segmentation voxels from the CSF segmentation. We then computed a measure of whole-brain CSF volume (mm^3) from the segmentation.* ***(C)*** *We observed a significant association between years since motor neuron disease diagnosis and total volume of CSF (r_s=0.83, p=0.01), though not when P9 was included (r_s=0.41, p=0.2).* ***(D)*** *A visualization of one participant's segmentation of the neurovasculture overlaid on the T1w MRI, the raw vasculature segmentation in 3D space and the manually cleaned segmentation of the SSS.* ***(E)*** *Using the SSS segmentation, the diameter of the SSS was measured to be on average 7.1 ± 0.9 mm across Stentrode implanted participants. Individual red circles reflect the average SSS width for the points where the Stentrode is implanted.*

medRxiv preprint doi: https://doi.org/10.1101/2025.09.19.25335875; this version posted September 25, 2025. The copyright holder for this preprint (which was not certified by peer review) is the author/funder, who has granted medRxiv a license to display the preprint in perpetuity.

It is made available under a CC-BY 4.0 International license .

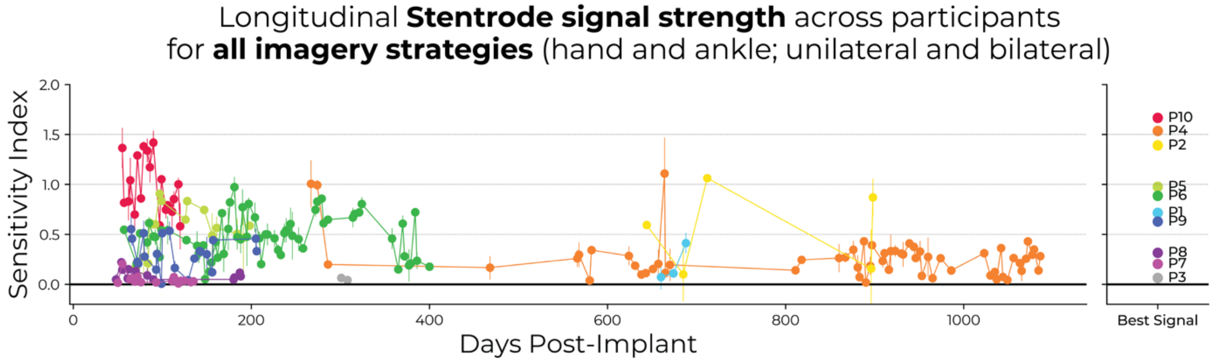

Supp. Figure 4. Stentrode motor signal strength datasets for all imagery strategies. *While the data plotted in Figure 5C is only for the preferred imagery strategy of each participant (either hand or ankle), the data displayed here includes all imagery strategies.*

medRxiv preprint doi: https://doi.org/10.1101/2025.09.19.25335875; this version posted September 25, 2025. The copyright holder for this preprint (which was not certified by peer review) is the author/funder, who has granted medRxiv a license to display the preprint in perpetuity.

It is made available under a CC-BY 4.0 International license .

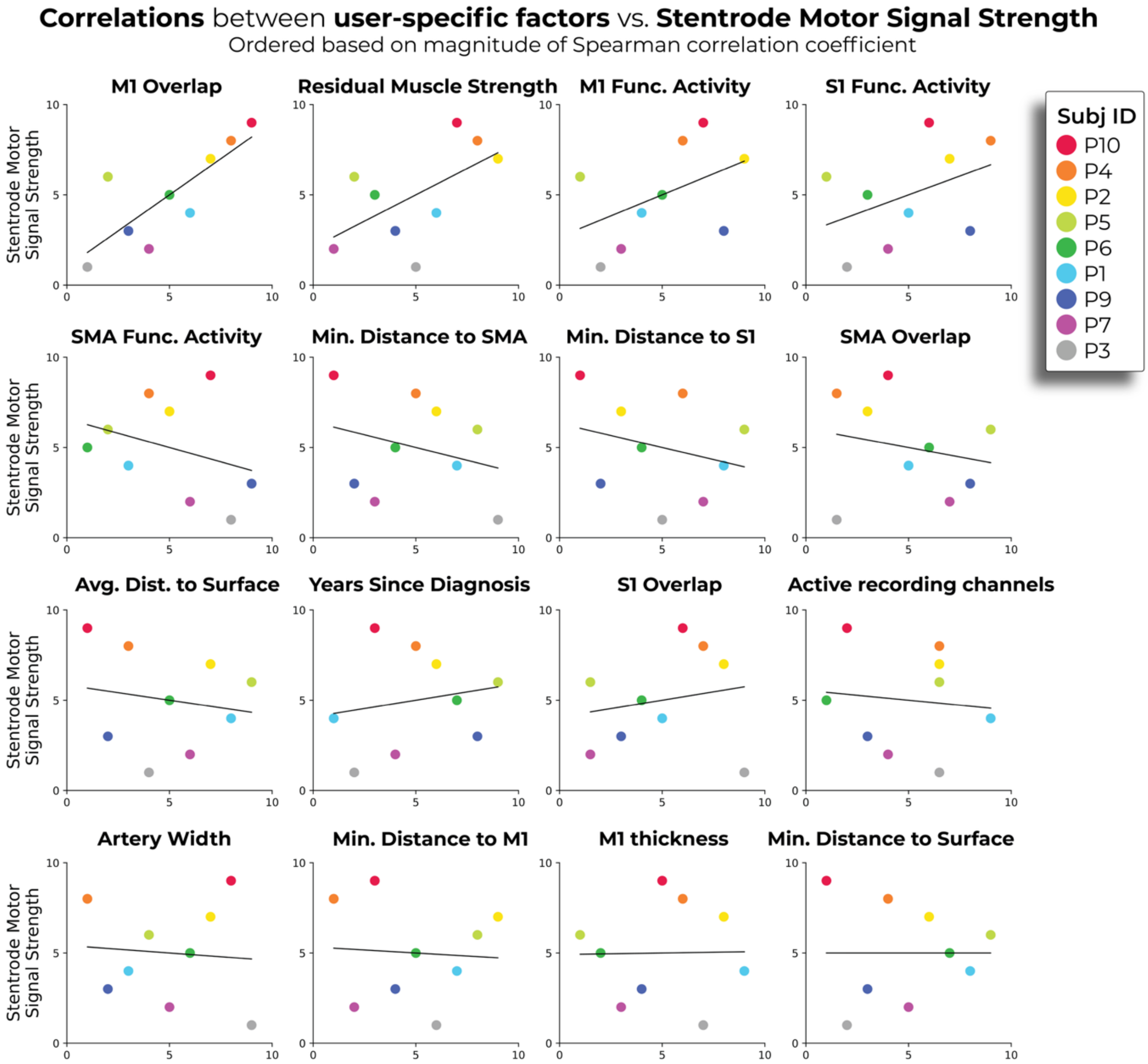

Supp. Figure 5. Plotting correlations between all user-specific factors and Stentrode motor signal strength. *All plotted data is the ranked values.*

medRxiv preprint doi: https://doi.org/10.1101/2025.09.19.25335875; this version posted September 25, 2025. The copyright holder for this preprint (which was not certified by peer review) is the author/funder, who has granted medRxiv a license to display the preprint in perpetuity.

It is made available under a CC-BY 4.0 International license .

Methods

Clinical trials and participant recruitment

Study participants were involved in 1 of 2 clinical trials: SWITCH or COMMAND. The SWITCH trial (n=4; participants P1 – P4; Clinicaltrials.gov: NCT03834857) was approved by the Human Research Ethics Committees of St. Vincent's Hospital, Royal Melbourne Hospital and Calvary Health Care Bethlehem in Australia. The clinical protocol for this trial has been previously published[10,11]. The COMMAND early feasibility trial (n=6; participants P4 – P10; Clinicaltrials.gov: NCT05035823) was conducted under an Investigational Device Exemption from the U.S. Food and Drug Administration and approved by the Institutional Review Boards at Western-Copernicus Group (1347924). Informed consent was obtained before any study procedures were conducted and in accordance with the Declaration of Helsinki.

Stentrode device and deployment

The Stentrode (Synchron, USA) consists of 16 platinum electrodes, each with a 300 μm diameter, mounted on a self-expanding nitinol scaffold measuring 8 × 40 mm. The inter-elctrode spacing is approximately 3mm. The Stentrode is connected to a 50cm flexible endovascular lead and inserted into an inductively powered internal telemetry unit[11] (ITU; Synchron, USA). The device, surgical deployment procedure and signal acquisition have been previously described in detail[10,11]. In the SWITCH trial, a common reference channel was fixed to an electrode located on the ITU. In the COMMAND trial, the reference channel was configurable across one of the 16 channels or the electrode on the IRTU.

Participants

Stentrode BCI group

The Stentrode BCI group included ten participants [two females; mean age ± STD = 58.4 ± 12.6]. Eight participants were diagnosed with ALS, one participant diagnosed with PLS (P4), and one participant diagnosed with an arterial ischemic stroke in the brainstem (P8; see Table 1 and Supp. Table 1 for all participant demographics).

medRxiv preprint doi: https://doi.org/10.1101/2025.09.19.25335875; this version posted September 25, 2025. The copyright holder for this preprint (which was not certified by peer review) is the author/funder, who has granted medRxiv a license to display the preprint in perpetuity.

It is made available under a CC-BY 4.0 International license .

Participant ID	***Baseline Functional Status***
P1	Limited mobility of upper and lower body starting out, eventually developed pseudo-bulbar issues (e.g., intense coughing).
P2	Slow progressive condition; upper body started losing functionality and very slowly affected his lower limbs, causing him to be wheelchair bound. Breathing difficulties made it difficult for him to speak and made him tire easily.
P3	Fast progressing condition; affected his whole body.
P4	Slow progressing condition; limited movement of lower limb, but proficient upper limb movement. Had an injured right shoulder making him unable to raise hand above shoulder level.
P5	Locked in; mechanically ventilated via a tracheostomy to breathe, only able to slightly move left side of mouth to indicate 'no' and looks up to indicate 'yes'.
P6	Locked in; mechanically ventilated via a tracheostomy to breathe, only able to slightly smile and very slight movement of right index finger.
P7	Locked in; mechanically ventilated via a tracheostomy to breathe, no residual movement.
P8	Room air via a tracheostomy to breathe; can make noises with passy-muir valve; residual movement in left hand; looks up and down to indicate 'yes' and 'no'
P9	Used supplemental oxygen and a gastrostomy feeding tube; could blink and slightly smile; could make slight noises to indicate distress
P10	Man-in-the-barrel ALS; minimal control of upper limbs; ambulatory and verbal.

Supplementary Table 1. Qualitative description of the functional status of participants.

Control group

The control group included 16 participants [9 females; mean age ± STD = 53.1 ± 6.37; all right-handed]. The only datasets analyzed from these participants were their structural T1w MRI scans (acquired from a publically available dataset; see ref.[28] for additional information about these participants, including MR scanning procedures). Importantly, there were no significant differences between the Stentrode BCI group (with motor neuron diseases) and the control group in age (Mann-Whitney independent samples test: W=109.0, p=0.13) or sex [though trending; Chi-Squared test: $X_2(1)$=3.3, p=0.06].

Manual muscle strength testing

Prior to implantation, participants underwent manual muscle strength testing. This involved a neurologist or physiatrist grading muscular contractions for different movements. For SWITCH trial participants, the following movements were assessed: fingers, wrist, elbow, shoulder, hip, knee, ankle, plantar flexion and dorsiflexion. For COMMAND trial participants, the following movements were assessed: finger abduction (pinky), middle finger flexion, wrist extension, elbow flexion, elbow extension, hip flexion, knee extension, ankle dorsiflexion, ankle plantar flexion and big toe dorsiflexion. For all participants, movements were assessed separately for the left and right sides of the body using the MRC muscle power scale[16,17], ranging from 0 to 5. The scores reflect the following: 0 = no muscular contraction; 1 = visible muscle contraction, but no movement at joint; 2 = movement at the joint, but not against gravity; 3 = movement against gravity, but not against resistance; 4 = movement against resistance, but less than full; 5 = movement against full resistance, normal strength. From this data, a bilateral whole-body compound score was generated. Scores were averaged for each body-part across sides (left, right) first and then averaged across body-parts, resulting in a measure of manual muscle strength, which we refer to as residual muscle strength.

Pre-implant MRI overview

Prior to implantation, all participants underwent an MRI session which included structural and functional imaging. Relevant for the present investigation, all participants underwent functional motor mapping scans and a T1w structural scan with a contrast agent.

medRxiv preprint doi: https://doi.org/10.1101/2025.09.19.25335875; this version posted September 25, 2025. The copyright holder for this preprint (which was not certified by peer review) is the author/funder, who has granted medRxiv a license to display the preprint in perpetuity.

It is made available under a CC-BY 4.0 International license .

Functional motor mapping

To assess whether participants could activate cortical motor networks during attempted movement, all participants underwent functional MRI scans while attempting to perform one or more movements of individual body-parts including: right ankle, left ankle, both ankles or both hands. For ankle movements, participants were instructed to repeatedly flex and extend their ankles. For hand movements, participants were instructed to repeatedly open and close their fingers. Importantly, the body-parts tested varied across participants, due to site-specific modifications to the imaging protocol catered to each participant. To guide participants' movements during scanning, instructions were delivered using audio- (i.e., Avotec MR-compatible headphones) and/or text-cues, displayed via a visual display projected into the scanner bore. The method for delivering cues (audio, text or both) varied across participants, due to varying technical capacities at each scan site.

The functional task design was structured in a block-design, where each block consisted of a 15s OFF ("REST") period followed by a 15s ON ("MOVE") period. The number of block repetitions varied between 8 – 10 across participants. The following participants performed 8 blocks: P1, P2, P3, P4, P6, P7, P10, followed by a 15s rest period. P8 performed 9 blocks followed by a 15s rest period. P5 and P9 performed 10 blocks. Finally, the number of functional runs varied from 1 – 4 depending on participants. For a detailed breakdown of the body-parts and number of functional runs tested per participant, see Supp. Table 2 below.

medRxiv preprint doi: https://doi.org/10.1101/2025.09.19.25335875; this version posted September 25, 2025. The copyright holder for this preprint (which was not certified by peer review) is the author/funder, who has granted medRxiv a license to display the preprint in perpetuity.

It is made available under a CC-BY 4.0 International license .

ID	MR Scanner; RF Coil; Site Location	Structural MRI (T1 MPRAGE)	Functional MRI (BOLD)	Body-parts tested (# of functional runs; # of motion outlier TRs > .9mm)
P1	• 3T Siemens Prisma<br>• 32Ch<br>• Melbourne, AUS	TR=1900ms<br>TE=2.34ms<br>TI=900ms<br>FA=8°<br>VoxelSize=0.9x0.9x1mm<br>FOV=288x288mm<br>Slices=208<br>Slice thickness=1.0mm	TR=2500ms<br>TE=30ms<br>Volumes=85<br>FA=90°<br>VoxelSize=3.75x3.75mm<br>FOV=64 x 64 mm<br>Slices=34<br>Slice thickness=3.0mm	• L ankle (1 run; 7)<br>• R ankle (1 run; 7)
P2	…	…	…	• L ankle (1 run; 0)<br>• R ankle (1 run; 0)
P3	…	…	…	• L ankle (1 run; 0)<br>• R ankle (1 run; 0)
P4	…	…	…	• L ankle (1 run; 0)<br>• R ankle (1 run; 3)
P5	• 3T GE SIGNA Architect<br>• 32Ch<br>• New York, USA	TR=8412ms<br>TE=3.168ms<br>TI=1100ms<br>FA=8°<br>VoxelSize=1mm^3<br>FOV=256x256mm<br>Slices=<br>Slice thickness=1mm	TR=3000ms<br>TE=60ms<br>Volumes=100<br>FA=90°<br>VoxelSize=3.75x3.75mm<br>FOV=64x64mm<br>Slices=<br>Slice thickness=4.0mm	• Both ankles (1 run; 0)
P6	• 3T GE SIGNA Architect<br>• 32Ch<br>• New York, USA	TR=8412ms<br>TE=3.168ms<br>TI=1100ms<br>FA=8°<br>VoxelSize=1mm^3<br>FOV=256x256mm<br>Slices=194<br>Slice thickness=1mm	TR=3000ms<br>TE=60ms<br>Volumes=85<br>FA=90°<br>VoxelSize=3.75x3.75mm<br>FOV=64x64mm<br>Slices=35<br>Slice thickness=4.0mm	• L ankle (1 run; 1)<br>• R ankle (1 run; 2)<br>• Both ankles (2 runs; run1: 1; run2: 1)<br>• Both hands (4 runs; run1: 1; run2: 3; run3: 3; run4: 4)
P7	• 3T Siemens Magnetom Biograph mMR<br>• 32Ch<br>• Pittsburgh, USA	TR=1900ms<br>TE=2.34ms<br>TI=900ms<br>FA=8°<br>VoxelSize=0.9mm^3<br>FOV=256x256mm<br>Slices=208<br>Slice thickness=0.9mm	TR=2500ms<br>TE=30ms<br>Volumes=85<br>FA=90°<br>VoxelSize=2mm^3<br>FOV= 110x110mm<br>Slices=32<br>Slice thickness=2.0mm	• L ankle (1 run; 0)<br>• R ankle (1 run; 0)
P8	• 3T Philips Igenia Elition X<br>• 32Ch<br>• Buffalo, USA	TR=7599ms<br>TE=3.4ms<br>TI=900ms<br>FA=8°<br>VoxelSize=0.3x0.3mm<br>FOV=240x240mm<br>Slices=170<br>Slice thickness=1.0mm	TR=3000ms<br>TE=35ms<br>Volumes=95<br>FA=90°<br>VoxelSize=1.79x1.79mm<br>FOV=96x94mm<br>Slices=36<br>Slice thickness=4.0mm	• Both hands (1 run; 0)<br>• Both ankles (2 runs; run1: 6; run2: 9)
P9	• 3T Philips Igenia Elition X<br>• 32Ch<br>• Buffalo, USA	TR=7599ms<br>TE=3.4ms<br>TI=900ms<br>FA=8°<br>VoxelSize=0.3x0.3mm<br>FOV=240x240mm<br>Slices=170<br>Slice thickness=1.0mm	TR=3000ms<br>TE=35ms<br>Volumes=100<br>FA=90°<br>VoxelSize=1.79x1.79mm<br>FOV=96x94mm<br>Slices=36<br>Slice thickness=4.0mm	• Both hands (1 run; 6)<br>• Both ankles (2 runs; run1: 32; run2: 3)
P10	• 3T Siemens Magnetom Biograph mMR<br>• 32Ch<br>• Pittsburgh, USA	TR=1900ms<br>TE=2.34ms<br>TI=900ms<br>FA=8°<br>VoxelSize=0.9mm^3<br>FOV=256x256mm<br>Slices=208<br>Slice thickness=0.9mm	TR=2500ms<br>TE=30ms<br>Volumes=85<br>FA=90°<br>VoxelSize=2mm^3<br>FOV=110x110mm<br>Slices=32<br>Slice thickness=2.0mm	• L ankle (1 run; 0)<br>• R ankle (1 run; 0)

***Supplementary Table 2. MRI scanning information for participants**. L=left; R=right; FA=flip angle; FOV=field of view; TR=repetition time; TE=echo time; TI=inversion time;* "**…**" = same as above.

medRxiv preprint doi: https://doi.org/10.1101/2025.09.19.25335875; this version posted September 25, 2025. The copyright holder for this preprint (which was not certified by peer review) is the author/funder, who has granted medRxiv a license to display the preprint in perpetuity.

It is made available under a CC-BY 4.0 International license .

MRI data acquisition

Participants were scanned at 1 of 4 MRI sites based in either: Melbourne (AUS), New York (USA), Buffalo (USA) or Pittsburgh (USA). As such, the scanner, head coil, structural- and functional MRI parameters vary across sites (for a breadown, see Supp. Table 2). MRI images, for all participants, were obtained using a 3-Tesla scanner. Prior to running a structural MR scan, participants underwent a time-resolved angiography with interleaved stochastic trajectories (TWIST) scan whereby a multihance contrast agent was administered. Although the data from this sequence were not used in the current investigation, a subsequent structural anatomical scan was performed using a T1-weighted magnetization prepared rapid acquisition gradient echo sequence (MRPAGE) in the sagittal plane with residual contrast agent from the TWIST angiography. The structural scan sequence parameters vary across participants, described in detail in Supp. Table 2. Next, functional data, based on the blood oxygenation level-dependent (BOLD) signal, were acquired using a gradient echo-planar imaging (EPI) sequence which included slices (32 or 34) with a slice thickness of either 2.0mm or 3.0mm, a repetition time (TR) of either 2500ms or 3000ms, and an echo time (TE) of 30ms. All other scan parameters are described in Supp. Table 2. The motor fMRI scans had either 85, 95 or 100 volumes, depending on the participant (approximately 5 minutes).

fMRI analysis

Functional MRI data were processed by using FMRIB's Expert Analysis Tool (FEAT; Version 6.0), part of FSL (FMRIB's Software Library, www.fmrib.ox.ac.uk/fsl), in combination with custom bash, Python (version 3) and MATLAB scripts (R2019b, v9.7, The Mathworks Inc, Natick, MA). Cortical surface reconstructions were produced using FreeSurfer [v. 7.1.1[45,46]] and Connectome Workbench (humanconnectome.org) software.

fMRI preprocessing

The following pre-statistical processing was applied: motion correction using MCFLIRT[47], non-brain removal using BET[48], spatial smoothing using a Gaussian kernel of FWHM 5mm for the functional task data, grand-mean intensity normalization of the entire 4D dataset by a single multiplicative factor, and high-pass temporal filtering (Gaussian-weighted least-squares straight line fitting, with σ = 100 s). Time-series statistical analysis was carried out using FILM with local autocorrelation correction[49]. The time series model included trial onsets convolved with a double γ HRF function; six motion parameters were added as confound regressors. Indicator functions were added to model out single volumes identified to have excessive motion (>0.9 mm). A separate regressor was used for each high motion volume (deviating more than .9mm from the mean position). For the motor mapping scans, the median number of outlier volumes for an individual scan, across all participants, was 1 volume (range: 0 – 32).

Low level task-based analysis

We applied a general linear model (GLM) using FEAT to each functional run. Each movement's task data was modeled against rest resulting in z-statistic maps, where each voxel represents the statistical significance of functional activity relative to rest, with the results thresholded for significance. As previously described, the number of runs performed varied between 1 - 4 across participants, for a given task. For participants with just 1 run of a task, the resulting z-stats were registered to the participant's structural T1w image. For participants with multiple runs of a single task, estimates from the individual runs were averaged in a voxel-wise manner using a fixed effects model with a cluster forming z-threshold of 2.3 and a family-wise error corrected cluster significance threshold of $p < 0.05$. Run-averaged z-stats were registed to the participant's structural T1w scan using FLIRT.

medRxiv preprint doi: https://doi.org/10.1101/2025.09.19.25335875; this version posted September 25, 2025. The copyright holder for this preprint (which was not certified by peer review) is the author/funder, who has granted medRxiv a license to display the preprint in perpetuity.

It is made available under a CC-BY 4.0 International license .

Cortical surface reconstruction

Structural T1w images were used to reconstruct the pial and white-gray matter surfaces using Freesurfer's recon-all command (https://surfer.nmr.mgh.harvard.edu/fswiki/recon-all). The grey and white matter segmentations of the reconstructions were then visually inspected and manually edited to correct any large geometric inaccuracies (for more information on the method for performing manual corrections of cortical surface reconstructions, please see https://surfer.nmr.mgh.harvard.edu/fswiki/FsTutorial/PialEdits_freeview). Surface co-registration across hemispheres and participants was done using spherical alignment. Individual surfaces were nonlinearly fitted to a template cortical surface, first in terms of the sulcal depth map, and then in terms of the local curvature, resulting in an overlap of the fundus of the central sulcus across participants[50].

Mapping functional activity onto the cortical surface

For surface-based analyses, functional mapping data, already registered to the structural T1w, were then projected onto the cortical surface using the workbench command's *volume-to-surface-mapping* function which included a ribbon-constrained mapping method. One important consideration is that different tasks were attempted across participants. Therefore, for scans where a single ankle was moved (right or left ankle), we opted to map the activity for only the contralateral hemisphere, as displayed in Figure 2. Alternatively, for scans involving moving both ankles, activity for each hemisphere (where a single hemisphere activity would include both the ipsi- and contra- activations) were mapped onto the cortical surface.

Regions of interest

Defined regions of interest (ROIs) were generated using one of two approaches: surface-to-surface mapping of the Glasser Human Connectome Project parcellation atlas[51] and (2) using probabilistic cytoarchitectonic maps[50]. From the Glasser atlas, the following regions were mapped (using *mri_surf2surf*) onto each participant's cortical surface: M1 (BA4), S1 (BA3b) and medially extended S1 (BA3b, BA5m, BA5mv, BA1). For the univariate ROI analysis, we were interested in activity directly under the SSS. Therefore, we further restricted the left- and right-hemisphere M1 and S1 ROIs to their most medial portions. We also defined a SMA region, which does not have strict anatomical boundaries[52]. Considering the Glasser atlas defines multiple sub-regions on the medial surface, just rostral of M1 [the medial caudal portion of BA6, superior frontal language area (SFL), and supplementary and cingulate eye field (SCEF)], we opted to use the SMA (pre-SMA) region boundaries used in ref[53], defined using probabilistic cytoarchitectonic maps on the group average cortical surface. These boundaries were then projected onto each participant's cortical surface. Additionally, as some ROI analyses were performed on the volume, these ROIs were mapped to the volume, using freesurfer's label-to-volume-mapping method with the ribbon-constrained option.

As a supplementary analysis for P5, we generated the boundaries of the precentral gyrus using the Desiken-Killiany Atlas (https://surfer.nmr.mgh.harvard.edu/fswiki/CorticalParcellation), default to freesurfer. This was displayed in Supp. Figure 1C.

Univariate analyses

Visualizing activation maps

As previously described, for each participant, statistically thresholded ankle activity were mapped onto the cortical surface. In Figure 2A, these activation maps were displayed with a common minimum statistical threshold ($Z > 2.3$). As a supplementary analysis for 3 participants that underwent additional scans when attempting to move their hands, we displayed the ankle activation maps alongside the hand activation maps (Supp. Figure 1C). Next, we generated a group-level ankle movement activation map, the individual participant activation maps (z-stats)

medRxiv preprint doi: https://doi.org/10.1101/2025.09.19.25335875; this version posted September 25, 2025. The copyright holder for this preprint (which was not certified by peer review) is the author/funder, who has granted medRxiv a license to display the preprint in perpetuity.

It is made available under a CC-BY 4.0 International license .

were averaged in a voxel-wise manner. This approach was taken, as opposed to using a mixed-effects model, due to the task differences across participants. The resulting group average z-stat was then mapped onto a standard cortical surface. This activity was minimally thresholded (Z > 2.3) and displayed on a standard pial cortical surface (Figure 2B).

Activity within regions of interest

Using the SMA ROI and the medially constrained M1 and S1 ROIs, the average z-stat within each region was extracted for each participant (Figure 2C). Additionally, to estimate the spatial coverage of activity within the regions, the number of significantly activated voxels (Z > 2.3) was computed (Supp. Figure 1B).

Generating a superior sagittal sinus segmentation

In the investigation, one feature we aimed to extract is spatial information about the SSS. To generate this, we segmented out the neurovasculature in 3D Slicer (https://www.slicer.org/). This was performed using a pipeline that leveraged a Vesselness filtering module, comprehensively described at https://github.com/lassoan/SlicerSegmentationRecipes. The resulting segmentation was then displayed on its own and edited manually to remove secondary vessels. The resulting segmentation was then saved as a .STL file (see Supp. Figure 2).

Determining the location of the Stentrode BCI

Using the post-implant CT image to isolate the Stentrode

All participants underwent post-implant CT scans to determine the position of the Stentrode BCI. CT scans were taken 3-months after implantation. Each participant's CT image was registered to their structural T1w MRI scan using FSL's FLIRT (FMRIB's Linear Image Registration Tool). To ensure the Stentrode image was not atypically morphed, a rigid-body registration was performed (6 degrees of freedom) using the mutual information cost function. Finally, the search range for the rotation parameters were set to [-180°, 180°] along the x, y, and z axes. To extract the location of the Stentrode, the CT-registered image was loaded into 3D Slicer. On a slice-by-slice basis, the Stentrode was manually segmented out for each participant. The resulting segmentation was saved as an .STL file (see Supp. Figure 2).

Next, we displayed the cortical surfaces and segmentations for the SSS and Stentrode in a common coordinate space (see example in Figure 3A). First, we modified the length of the manual Stentrode segmentation. Across participants, the resulting Stentrode segmentations ranged in length from 26.7 – 33.4mm. However, the exact distance of the Stentrode's most rostral electrode to the most caudal electrode is 25mm. Therefore, we restricted the length of each segmentation from the most rostral point of the segmentation back 25mm. Next, we aimed to generate a simplified approximation of the Stentrode position. We generated a straight line from the most rostral point of the segmentation to the most caudal point of the segmentation. We next wanted to curve this line and position it within the middle of the SSS. We used custom code written in MATLAB, whereby the rostral and caudal line endpoints were positioned in the center of their relative location in the SSS. The midpoint of the line was then similarly adjusted. A polynomial fit was then applied to the line, generating a curved path that approximated the Stentrode's real-world positioning within the SSS.

To generate the images in Figure 4 of the Stentrode BCI position with the cortical surfaces, a spatially accurate Stentrode model (black) was then aligned to the segmentation such that the most rostral segmentation point coincides with the most rostral point of the Stentrode model. The Stentrode model was then manually positioned to best fit the orientation of the manual segmentation.

medRxiv preprint doi: https://doi.org/10.1101/2025.09.19.25335875; this version posted September 25, 2025. The copyright holder for this preprint (which was not certified by peer review) is the author/funder, who has granted medRxiv a license to display the preprint in perpetuity.

It is made available under a CC-BY 4.0 International license .

Computing spatial distances

Using the 25mm curved Stentrode line, we defined a point along the line every 0.5mm. Then, we computed the distance from each point to the nearest cortical surface vertex in the left hemisphere and the right hemisphere (50 distance estimations per hemisphere). We displayed a visualization of this approach in Figure 3B. For each hemisphere separately, we computed 5 different distances: 1) the minimum distance of each of these projections to either the left or right hemisphere (i.e., minimum distance to cortex), 2) the average distance to the cortical surface, averaging across the lines for each hemisphere separately (i.e., average distance to cortex), 3) the minimum distance to M1, 4) S1, and 5) SMA. We averaged the distances for each hemisphere to get 1 value per participant. Finally, the width of the SSS was also computed. This was performed by restricting the SSS segmentation to just what was in-line with the Stentrode line and computing multiple distance estimations across the full extent of the SSS segmentation (an example displayed in Supp. Figure 3A).

Percent overlap of Stentrode and brain regions

To compute a measure of overlap between the Stentrode and brain regions (SMA, M1, S1 etc.), we defined the most rostral point of each region on the medial surface, for each hemisphere separately. A 2-D coronal plane was generated at each point, such that it cut through the Stentrode segmentation. Considering the brain regions are spatially continuous along the medial wall, the boundaries of each region ran from the most rostral point of each region to the most rostral point of the subsequent caudal region (e.g., SMA = rostral of SMA to rostral of M1). The percent overlap values were computed by calculating the distance of Stentrode overlapping each region and dividing these values by the total distance of the Stentrode (25mm). We averaged the percent overlap values across hemispheres to get 1 value per region.

Cortical thickness

After the Glasser Atlas'[51] M1 and S1 boundaries were mapped to each participant's pial cortical surfaces (using a ribbon-constrained manner), the masks were visualized on both the surface and volume to ensure they were spatially accurate (i.e., only included the grey matter). To compute cortical thickness, we implemented the *mri_segstats* command to compute the cortical thickness for each region and hemisphere. We averaged the cortical thickness values across hemispheres to get 1 value per region.

Computing the volume of the cerebrospinal fluid

To investigate whether the amount of CSF increases with cortical atrophy, we aimed to compute a measure of CSF volume. We used FreeSurfer's *mri_segstats* tool to extract total volumes of the CSF, grey matter, white matter and subcortical structures (visualized in Supp. Figure 3B for 1 participant). We then subtracted the SSS segmentation for each participant from the CSF segmentation to yield an adjusted intracranial CSF estimate excluding the primary venous structure.

Computing a measure of Stentrode motor signal strength

Motor signal tests

Throughout both clinical trials, participant engaged in a task to test their ability to generate a click using their Stentrode BCI. The task involved refraining from making a click for 10s (rest period) and attempting the corresponding movement on the screen within 10s (go period), guided by visual cues. There were 10 trials per CCT run and every run finished with an additional 10s rest period (for a breakdown of the number of sessions collected see Supp. Table 3 below). All tests were conducted within the home of each participant.

medRxiv preprint doi: https://doi.org/10.1101/2025.09.19.25335875; this version posted September 25, 2025. The copyright holder for this preprint (which was not certified by peer review) is the author/funder, who has granted medRxiv a license to display the preprint in perpetuity.

It is made available under a CC-BY 4.0 International license .

Subj ID	Body-part tested (number of sessions)	Average number of blocks per session
P1	**Left ankle** (3)	2.33
P2	**Both ankles** (1), left ankle (4)	2.20
P3	**Right ankle** (2)	1
P4	**Both ankles** (58), left ankle (2)	2.25
P5	**Both ankles** (18)	2.44
P6	Both ankles (39), left hand (1), **right hand** (22)	2.38
P7	**Both ankles** (10), right ankle (1), both hands (1), right hand (3), right hip (1), mouth (2), left shoulder (1)	4.73
P8	**Both ankles** (13), left hand (1)	4.62
P9	Both ankles (4), **right hand** (20)	2.27
P10	Both ankles (1), **right hand** (19)	3.26

Supp. Table 3. Number of Stentrode motor signal test sessions and body-parts tested across participants. *The bolded body-part is the preferred body-part for Stentrode control.*

Sensitivity index

The sensitivity index was calculated in the following steps. For each participant, channels with high impedance values (>700 kOhms) and exhibiting signs of erratic pertubations via visual inspection were omitted. Data from the remaining channels were bandpass filtered at 100-200 Hz with a 3rd order Butterworth infinite impulse response filter, based on a previous study showing that movement-related modulation in Stentrode recordings is most prominent within this frequency range[14]. Instantaneous power within 100ms non-overlapping windows were derived by calculating the log of the variance per channel, then the values were averaged across the channels. The sensitivity index (d') was calculated between all rest periods and go periods, where:

$$d' = 2\frac{\mu_{go\ period} - \mu_{rest\ period}}{\sigma_{go\ period} + \sigma_{rest\ period}}$$

And μ and σ denote the mean and the standard deviation of each period. For each participant, their highest sensitivity index value was then used as their Stentrode motor signal strength value.

Statistical analyses

All statistical analyses were performed using either Python scripts utilizing scipy.stats and statsmodels.stats.multitest or JASP (0.17.2.1). Tests for normality were conducted using a Shapiro–Wilk test. Though, due to the small number of samples (n=10), we opted to primarily use non-parametric statistics in most instances. For the functional neuroimaging data, when comparing whether 1 region had more activity than another region, we used a paired Wilcoxon-signed rank test. For the structural neuroimaging data, when comparing cortical thickness measures between regions (M1, S1) and groups (motor neuron disease, controls), we used a repeated measures ANOVA with region as a within-subject factor and group as the between-subject factor. Post-hoc group comparisons were performed with non-parametric Mann Whitney U Tests where p-values were corrected for 2 comparisons. Additionally, when comparing demographics between the Stentrode participants with motor neuron diseases and the control participants, a Mann Whitney U-test was used to test for differences in age and a chi-squared test was used to test for differences in sex. For the Stentrode motor signal strength data, for each participant, we tested whether values were significantly greater than 0 using a Wilcoxon signed-rank test with 0 set as the test value. For the correlation analyses, we used non-parametric Spearman correlations. Finally, to investigate the relationship between selected

medRxiv preprint doi: https://doi.org/10.1101/2025.09.19.25335875; this version posted September 25, 2025. The copyright holder for this preprint (which was not certified by peer review) is the author/funder, who has granted medRxiv a license to display the preprint in perpetuity.

It is made available under a CC-BY 4.0 International license .

predictors and motor signal strength (sensitivity index), we performed a Lasso regression analysis. To standardize the data, we applied a standard scaling procedure to the predictor variables. The Lasso model was fitted with an alpha value of 0.1, which is a regularization parameter that controls the strength of the penalty on the coefficients. A threshold of 0.05 was used for statistical significance.
